# Strawberry Vein Banding Virus Movement Protein P1 Interacts With Light-Harvesting Complex II Type 1 Like of *Fragaria vesca* to Promote Viral Infection

**DOI:** 10.3389/fmicb.2022.884044

**Published:** 2022-05-26

**Authors:** Shiqiang Xu, Xiangxiang Zhang, Kai Xu, Zhanqi Wang, Xueping Zhou, Lei Jiang, Tong Jiang

**Affiliations:** ^1^Department of Plant Pathology, School of Plant Protection, Anhui Agricultural University, Hefei, China; ^2^Key Laboratory of Vector Biology and Pathogen Control of Zhejiang Province, College of Life Sciences, Huzhou University, Huzhou, China; ^3^State Key Laboratory for Biology of Plant Disease and Insect Pest, Institute of Plant Protection, China Academy of Agricultural Sciences, Beijing, China; ^4^Anhui Province Key Laboratory of Crop Integrated Pest Management, School of Plant Protection, Anhui Agricultural University, Hefei, China; ^5^Key Laboratory of Biology and Sustainable Management of Plant Diseases and Pests of Anhui Higher Education Institutes, School of Plant Protection, Anhui Agricultural University, Hefei, China

**Keywords:** strawberry vein banding virus, intercellular movement, FvLHC II-1L protein, P1 protein, viral infection

## Abstract

Chlorophyll a/b-binding protein of light-harvesting complex II type 1 like (LHC II-1L) is an essential component of photosynthesis, which mainly maintains the stability of the electron transport chain. However, how the LHC II-1L protein of *Fragaria vesca* (FvLHC II-1L) affects viral infection remains unclear. In this study, we demonstrated that the movement protein P1 of strawberry vein banding virus (SVBV P1) interacted with FvLHC II-1L *in vivo* and *in vitro* by bimolecular fluorescence complementation and pull-down assays. SVBV P1 was co-localized with FvLHC II-1L at the edge of epidermal cells of *Nicotiana benthamiana* leaves, and FvLHC II-1L protein expression was upregulated in SVBV-infected *F. vesca*. We also found that FvLHC II-1L effectively promoted SVBV P1 to compensate for the intercellular movement of movement-deficient potato virus X (PVX^ΔP25^) and the systemic movement of movement-deficient cucumber mosaic virus (CMV^ΔMP^). Transient overexpression of FvLHC II-1L and inoculation of an infectious clone of SVBV showed that the course of SVBV infection in *F. vesca* was accelerated. Collectively, the results showed that SVBV P1 protein can interact with FvLHC II-1L protein, which in turn promotes *F. vesca* infection by SVBV.

## Introduction

The strawberry vein banding virus (SVBV) is a latent virus that severely harms strawberry plants in major strawberry growing areas worldwide (Ratti et al., 2009; Chen et al., 2016a). In China, SVBV, which is primarily transmitted by aphids or tissue culture seedlings, is mainly distributed in the Sichuan, Hebei, Liaoning, Shandong, and Anhui provinces (Pan et al., 2018; Jiang et al., 2021), where SVBV-infected strawberries display characteristic symptoms, such as weak growth, uneven leaf color, a decrease in the number of creeping stems, and deformed fruits (Frazier, 1974).

Strawberry vein banding virus is a double-stranded DNA virus belonging to the genus *Caulimovirus* of the *Caulimoviridae* family. It contains seven open reading frames (ORFs), each of which encode a protein (Pattanaik et al., 2004). ORF I encodes a movement protein (MP) called P1 that can promote the intercellular movement of SVBV, which plays an essential role in the process of viral infection (Rui et al., 2021). SVBV shares similar genome structure with cauliflower mosaic virus (CaMV) belonging to the same genus. According to the proteins encoded by each ORF of CaMV, it was inferred that ORF II, III, IV, and V of SVBV may encode proteins associated with aphid infection, a DNA-binding protein, a coat protein (CP), a reverse transcriptase protein (Pan et al., 2018). P6, encoded by ORF VI, is a multifunctional protein that functions as an RNA silencing suppressor and translation transactivator (Li et al., 2018a), and ORF VII encodes an unknown protein (Schoelz et al., 2016). Our previous study showed that SVBV P1 may regulates intracellular and intercellular movement of SVBV during viral infection (Rui et al., 2021).

After invading the host plants, viruses spread through plasmodesmata (PD) to neighboring cells with the help of MPs (Schoelz et al., 2011). MPs can be transported intracellularly and distantly by binding virus particles, binding viral nucleic acids, inducing the formation of PD tubular structures, and changing the size of the PD aperture (Chen et al., 2000; Lucas, 2006; Park et al., 2014). It has been shown that host-encoded proteins interacting with viral MPs can control the pore size of PD and/or change the localization of viral MP on the PD to affect the process of viral infection (Peiro et al., 2014; Wu and Cheng, 2020). For example, the Alfalfa mosaic virus (AMV) MP can interact with *Arabidopsis thaliana* patellin 3 (AtPATL3); furthermore, overexpression of *AtPATL3* makes AMV MP unable to target PD, thereby affecting the intercellular movement of the virus (Peiro et al., 2014). Similarly, the rice membrane-associated protein remorin (REM1), which interacts with the MP NSvc4 encoded by the rice stripe virus (RSV), is involved in viral infection. Silencing of the *NbREM1* gene in *Nicotiana benthamiana* can reduce the deposition of callose, enhance the permeability of PD, and promote viral infection (Fu et al., 2018). Triple gene block protein 1, encoded by barley stripe mosaic virus (BSMV), can hijack the nucleolar protein fibrillarin 2 to form a viral ribonucleoprotein movement complex, thus promoting viral cell-to-cell movement (Li et al., 2018b). Moreover, *Citrus macrophylla* miraculin-like protein 2 is capable of hijacking MP P33 of Citrus tristeza virus to form aggregates in the cytoplasm and inhibit the cell-to-cell movement of the virus (Sun et al., 2021). Thus, host factors may interact with MPs to affect the process of viral infection.

In higher plants, photosystem II (PSII) is a large protein complex located on the thylakoid membrane of chloroplasts. It comprises the PSII core complex and light-harvesting complex II (LHC II), which drive water oxidation and are key components of photosynthesis (Huang et al., 2013). LHC II mainly binds to chlorophyll a/b and has three transmembrane domains, generally in the form of small monomers and macromeres (Neilson and Durnford, 2010). LHC II is responsible for transferring the absorbed light energy to the PSII reaction center and maintaining the stability of the PSII electron transport chain (Allen et al., 1981; Nilsson et al., 1997). LHC II not only participates in plant photosynthesis but also regulates plant growth and development. Silencing of *AtLhcb1* in *Arabidopsis thaliana* hindered the formation of LHC II trimers, affected photosynthesis, and caused plant dwarfing (Pietrzykowska et al., 2014); loss-of-function mutations in *AtTHF1* decreased the rate of LHC II degradation and delayed leaf yellowing (Huang et al., 2013). Our previous study indicated that SVBV P1 can interact with chlorophyll a/b-binding protein of LHC II type 1 like of *Fragaria vesca* (FvLHC II-1L), based on the results of yeast two-hybrid (Y2H) assay (Zhang et al., 2019), but the role of FvLHC II-1L in SVBV infection is still largely unknown.

In this study, we used bimolecular fluorescence complementation (BiFC) and pull-down assays to demonstrate that P1 interacts with FvLHC II-1L *in vivo* and *in vitro*. Quantitative real-time polymerase chain reaction (RT-qPCR) showed that FvLHC II-1L expression was significantly upregulated following SVBV infection. The subcellular localization assay revealed that SVBV P1 and FvLHC II-1L co-localized at the edge of the plant cell and that FvLHC II-1L was able to promote SVBV P1 aggregation. Furthermore, we showed that FvLHC II-1L promoted SVBV P1 to compensate for the movement of movement-deficient potato virus X (PVX^ΔP25^) and cucumber mosaic virus (CMV^ΔMP^). Overexpression of FvLHC II-1L enhanced SVBV infection in *F. vesca*.

## Materials and Methods

### Plant Materials, *Agrobacterium* and Vacuum Infiltration

All plants used in this study were grown in a growth chamber, set at 25°C, under 60% relative humidity and a 16 h light and 8 h dark photoperiod.

Six- to eight-week-old *N. benthamiana* plants were used for *Agrobacterium tumefaciens* infiltration, as described previously (Chen et al., 2016b). Equal volumes of individual *Agrobacterium* cultures (optical density at 600 nm, OD_600_ = 1.0) were mixed before co-infiltration.

Strawberry plants were infiltrated as described previously (Tian et al., 2015). The roots of strawberry plant seedlings were rinsed with distilled water, and then, whole plants were submerged in *A. tumefaciens* inoculum containing pTRV1 and pTRV2 or its derivatives (OD_600_ = 1.0) and placed in a vacuum at 101 kPa atmospheric pressure for 30 s; this procedure was repeated. The treated plants were washed with distilled water and cultured in pots containing nutrient solution, and after 24 h, they were transplanted into pots containing nutrient soil.

### BiFC Assay

The coding sequences of SVBV P1 (GenBank No: X97304.1) and FvLHC II-1L (GenBank No: XM_004303830.2) were cloned into pCV-cYFP and pCV-nYFP, respectively (Li et al., 2018a), to generate cYFP-SVBV P1, nYFP-FvLHC II-1L, nYFP-P1, and cYFP-FvLHC II-1L. The recombinant plasmids were transformed into *A. tumefaciens* (GV3101) and co-infiltrated into *N. benthamiana* leaves. The agro-infiltrated leaves were observed using confocal microscopy (Olympus FV1000, Tokyo, Japan) at approximately 3 days post inoculation (dpi).

### Pull-Down Assay

The full-length FvLHC II-1L or cyan fluorescent protein (CFP) respectively was inserted between the *BamH*I/*Sal*I restriction sites within the pCAM1307-3*Flag based expression vector to produce pCAM1307-FvLHC II-1L-3*Flag (FvLHC II-1L-Flag) or pCAM1307-CFP-3*Flag (CFP-Flag). To detect the SVBV P1–FvLHC II-1L interaction, a pull-down assay was conducted using protein extracts of FvLHC II-1L-Flag from infiltrated *N. benthamiana* leaves at 3 dpi. The full-length cDNAs of SVBV P1 were amplified and inserted into the pHMTc. The plasmid pHMTc was kindly supplied by Prof. Xiaorong Tao (Nanjing agricultural University, China). The construct pHMTc-SVBV P1 was transformed into *Escherichia coli* BL21 (DE3) cells. Total protein extracts were incubated with the maltose-binding protein (MBP)-SVBV P1 fusion proteins. The MBP pull-down assay was performed as previously described (Zhu et al., 2017).

### Subcellular Localization Assay

For the subcellular localization study, SVBV P1 and FvLHC II-1L were inserted into a modified pCAM2300 containing green fluorescent protein (GFP) or red fluorescent protein (RFP), respectively, and then transformed into *A. tumefaciens* (Feng et al., 2013). The cultures were co-infiltrated with *N. benthamiana*. After 3 days, the infiltrated leaves were imaged using a confocal microscope (Olympus FV1000, Tokyo, Japan). Excitation wavelength was 488 nm for GFP, 514 nm for yellow fluorescent protein (YFP), 561 nm for RFP, and 640 nm for chloroplast.

### RNA Extraction and RT-qPCR

Total RNA was extracted from agro-infiltrated leaves using the Omini Plant RNA Plant Kit (CWBIO, Beijing, China). cDNA was synthesized using PrimeScript^™^ RT reagent kit (TaKaRa, Tokyo, Japan). RT-qPCR was performed as described previously (Li et al., 2019).

### Protein Extraction and Western Blotting

Total protein was extracted from agro-infiltrated leaves using RIPA lysis buffer II (Sangon Biotech, Shanghai, China). After electrophoresis, the gel was placed in Coomassie Brilliant Blue stain (CBB, BeyoBlue^™^ Coomassie Blue Super Fast Staining Solution; Beyotime, Shanghai, China), soaked for 1 h, then decolorized with pure water. Western blotting was performed as previously described (Tian et al., 2014; Li et al., 2018a). Total protein was separated *via* sodium dodecyl sulfate–polyacrylamide gel electrophoresis, transferred to nitrocellulose membranes, then separately incubated using GFP mouse monoclonal antibody, c-Myc mouse monoclonal antibody, DYKDDDDK (Flag) mouse monoclonal antibody, MBP mouse monoclonal antibody (TransGen, Beijing, China), FvLHC II-1L rabbit polyclonal antibody, SVBV P1 rabbit polyclonal antibody, and then incubated using goat anti-rabbit IgG (H + L), HRP Conjugate and goat anti-mouse IgG (H + L), HRP Conjugate (TransGen, Beijing, China) with dilutions at 1:5,000 was used in immunoblot analysis. The detection signal was visualized using the EasySee Western Blot Kit (Transgene Biotech, Beijing, China).

### PVX Movement Complementation Experiment

For potato virus X movement complementation experiments, a movement-deficient PVX-GFP construct (PVX^ΔP25^-GFP) and construct P25, expressing the PVX P25 protein, were kindly supplied by Prof. Fei Yan (Ningbo University, China). The segment of FvLHC II-1L fused Myc-tag in the 5-termius was inserted between the *BamH*I/*Sal*I restriction sites within the pBin438 based expression vector to produce pBin438-Myc-FvLHC II-1L. The full-length SVBV P1 was inserted between the BamHI/SalI restriction sites within the pBin438 based expression vector to produce pBin438-SVBV P1, and the assays were performed as previously described (Yan et al., 2012; Rui et al., 2021). The GFP fluorescence of the infiltrated leaves was observed using a confocal microscope (Olympus FV1000, Tokyo, Japan).

### CMV Complementation Assay

The CMV complementation assay was performed as previously described (Shen et al., 2014). The full-length SVBV P1 was inserted between the *Nco*I/*Xba*I restriction sites within the pCB301-CMV RNA3^ΔMP^ based expression vector to produce pCB301-CMV RNA3^ΔMP^-SVBV P1. The plasmid pCB301-CMV RNA3^ΔMP^ was kindly supplied by Prof. Xiaorong Tao (Nanjing agriculture University, China). *A. tumefaciens* containing pCB301-CMV RNA3 or pCB301-CMV RNA3^ΔMP^-SVBV P1 was mixed with *A. tumefaciens* containing pCB301-CMV RNA1 and RNA2 in a 1:1:1 ratio.

### Tobacco Rattle Virus (TRV)-Based Gene Overexpression Assay

The full-length ORF of FvLHC II-1L was inserted behind the CP of the TRV2 vector as previously described (Tian et al., 2014). pTRV1 and pTRV2 or its derivatives were transformed, in order, into *A. tumefaciens* and co-transfected into *F*. *vesca* using a vacuum-infiltration method, as previously described (Tian et al., 2015).

### DNA Extraction and Southern Blot Analysis

Strawberry vein banding virus-infected strawberry leaves were harvested at 35 dpi. Total DNA was extracted from the strawberry leaves using the CTAB method. Southern blotting was performed as previously described (Feng et al., 2016). SVBV DNA accumulation in SVBV-infected plants was detected by digoxigenin-labeled probes from the 5-termius of SVBV *CP* gene using DIG High Prime DNA Labeling and Detection Starter Kit II (Roche, Basel, Switzerland) according to manufacturer instructions.

### Genetic Transformation of FvLHC II-1L in *Nicotiana benthamiana*

The full-length FvLHC II-1L was inserted between the *Xba*I/*Hind*III restriction sites within the pCAM1307-Myc based expression vector to produce pCAM1307-Myc-FvLHC II-1L. The plasmid was transformed into *A. tumefaciens* strain LBA4404. The transgenic *N. benthamiana* plants of T0 generation were obtained by leaf disc transformation method. The homozygous FvLHCII-1L transgenic *N. benthamiana* was obtained after screening. RT-qPCR and Western blot analyzes were used to detect the expression of FvLHC II-11L. The primers used for vector construction are listed in Supplementary Table S1.

## Results

### SVBV P1 Interacts With FvLHC II-1L *in vitro* and *in vivo*

**Figure 1:**
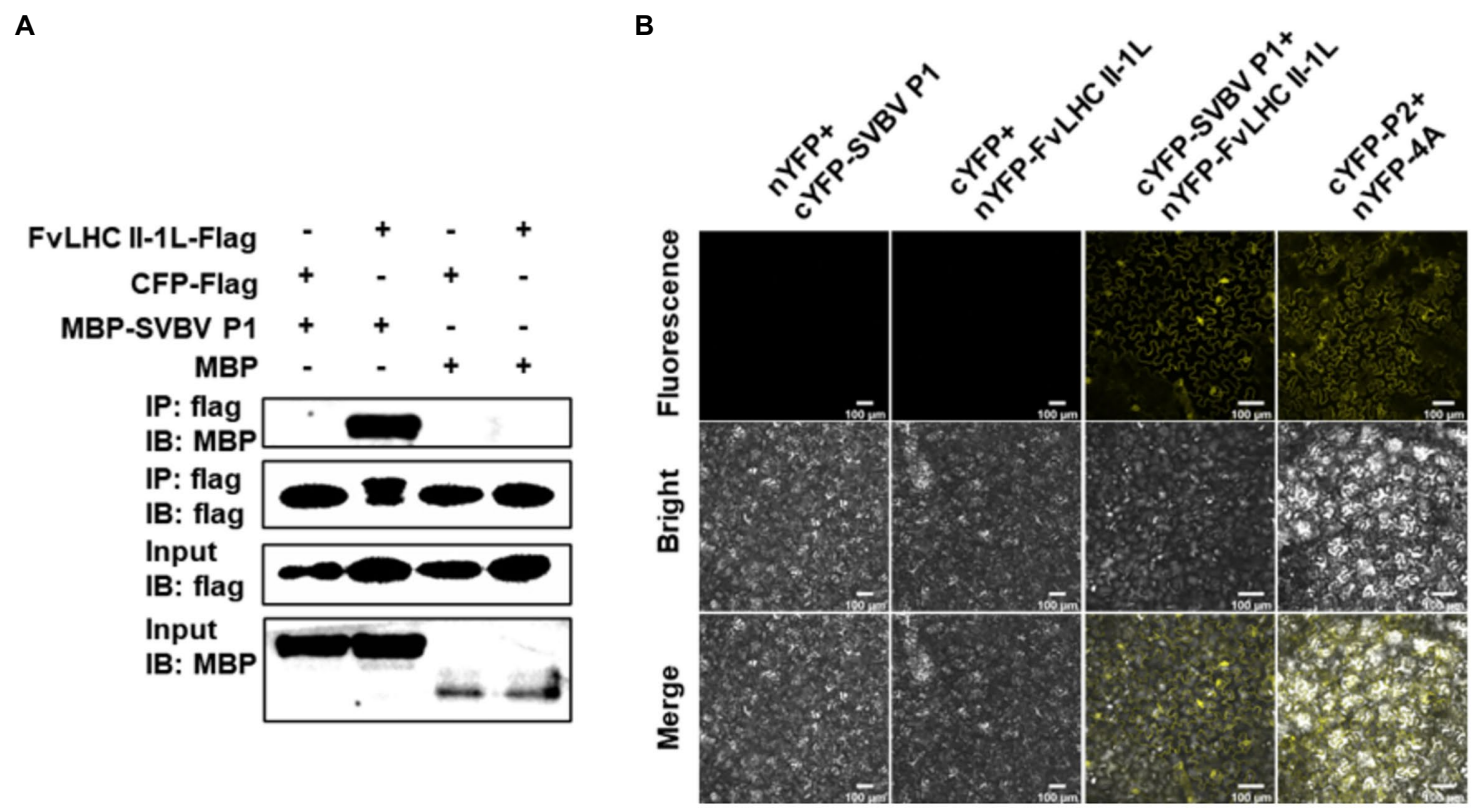
Strawberry vein banding virus (SVBV) P1 interacts with chlorohyll a/b-binding protein of light-harvesting complex II type 1 like of *Fragaria vesca* (FvLHC II-1L) *in vitro* and *in vivo*. **(A)** Interaction between SVBV P1 and FvLHC II-1L was elucidated using an *in vitro* pull-down assay. Maltose-binding protein (MBP)-SVBV P1 or MBP was expressed in, and purified from, *Escherichia coli* BL21(DE3). FvLHC II-1L-Flag or cyan fluorescent protein (CFP)-Flag was expressed in *Nicotiana benthamiana* leaves and harvested at 3 days post inoculation (dpi). Purified MBP-SVBV P1 or MBP was mixed with plant extract of FvLHC II-1L-Flag or CFP-Flag and pulled down with Flag beads. **(B)** Interaction between SVBV P1 and FvLHC II-1L was examined using an *in vivo* bimolecular fluorescence complementation (BiFC) assay. *N*. *benthamiana* leaves were co-agroinfiltrated with the following combinations of plasmids: cYFP-SVBV P1 and nYFP-FvLHC II-1L, nYFP-4A and cYFP-P2 (positive control), cYFP-SVBV P1 and nYFP, and nYFP-FvLHC II-1L and cYFP (negative controls). Yellow fluorescent protein (YFP) fluorescence was observed *via* confocal microscopy at 3 dpi. Bar scale = 100 μm.

To examine how SVBV P1 causes plant disease during viral infection, we screened a *F. vesca* cDNA library using the Y2H assay to identify the host proteins involved. Consequently, FvLHC II-1L was noted to interact with SVBV P1 (Zhang et al., 2019). To further verify the interaction between SVBV P1 and FvLHC II-1L, a pull-down experiment was performed. The results showed that MBP-SVBV P1 specifically pulled down the FvLHC II-1L-Flag protein (Figure 1A). SVBV P1 was absent in the cyan fluorescent protein (CFP)-Flag pulldown products, and FvLHC II-1L-Flag was not able to pull down MBP (Figure 1A), indicating that SVBV P1 can interact with FvLHC II-1L *in vitro*. Furthermore, a BiFC assay was performed to examine the interaction between SVBV P1 and FvLHC II-1L *in vivo* using *N. benthamiana*. As shown in Figure 1B, when cYFP-SVBV P1 and nYFP-FvLHC II-1l were co-expressed in *N. benthamiana* leaf cells, strong YFP fluorescence was observed, similar to the co-infiltration with nYFP-4A and cYFP-P2 (positive control). As expected, no fluorescence could be observed in *N. benthamiana* leaf epidermal cells co-infiltrated with cYFP-SVBV P1 and nYFP or nYFP-FvLHC II-1L and cYFP (negative controls; Figure 1B), indicating that SVBV P1 can interact with FvLHC II-1L *in vivo*. Collectively, these results suggest that SVBV P1 interacts with FvLHC II-1L *in vitro* and *in vivo*.

### Interaction Between SVBV P1 and FvLHC II-1L Alters Subcellular Localization of SVBV P1

**Figure 2:**
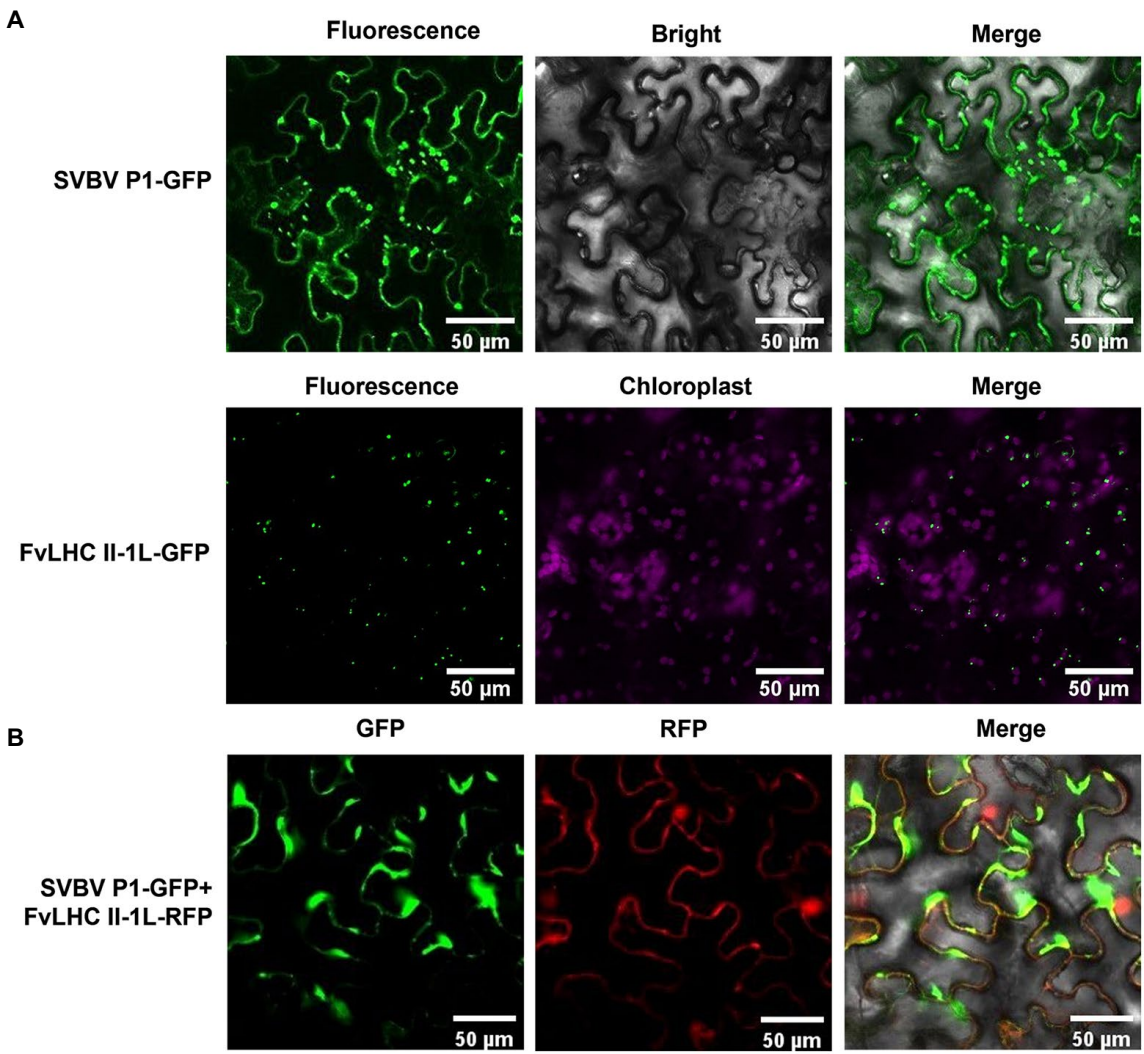
Subcellular localization of SVBV P1 and FvLHC II-1L. **(A)** Subcellular localization of SVBV P1 and FvLHC II-1L in *N. benthamiana* leaves. *N. benthamiana* leaves were infiltrated with the indicated vector combinations. Green fluorescent protein (GFP) was observed at 3 dpi. **(B)** The co-localization of SVBV P1 and FvLHC II-1L in *N. benthamiana* leaves. The fluorescence was observed *via* confocal microscopy at 3 dpi. Bar scale = 50 μm.

To investigate the subcellular localization of SVBV P1 and FvLHC II-1L, SVBV P1 and FvLHC II-1L were fused to the C-terminus of GFP, respectively, to obtain SVBV P1-GFP and FvLHC II-1L-GFP. The constructs were then transformed into *A. tumefaciens* and infiltrated into *N. benthamiana* leaves, as described previously (Rui et al., 2021). GFP fluorescence was observed in the cytoplasm and cell periphery of SVBV P1-GFP-infiltrated *N. benthamiana* leaves, and GFP fluorescence was observed in chloroplasts of FvLHC II-1L-GFP-infiltrated *N. benthamiana* leaves (Figure 2A). To determine whether FvLHC II-1L affects the localization of SVBV P1, we co-expressed SVBV P1-GFP and FvLHC II-1L-RFP in *N. benthamiana* leaves using the agroinfiltration method. The results showed that SVBV P1-GFP and FvLHC II-1L-RFP were co-localized at the edge of *N. benthamiana* epidermal cells, and GFP fluorescence converged at the edge of cells (Figure 2B). These results suggest that FvLHC II-1L promotes SVBV P1 aggregation at the periphery of plant cells.

### *FvLHC II-1L* Gene Expression IsUpregulated in SVBV-Infected Fragariavesca

**Figure 3:**
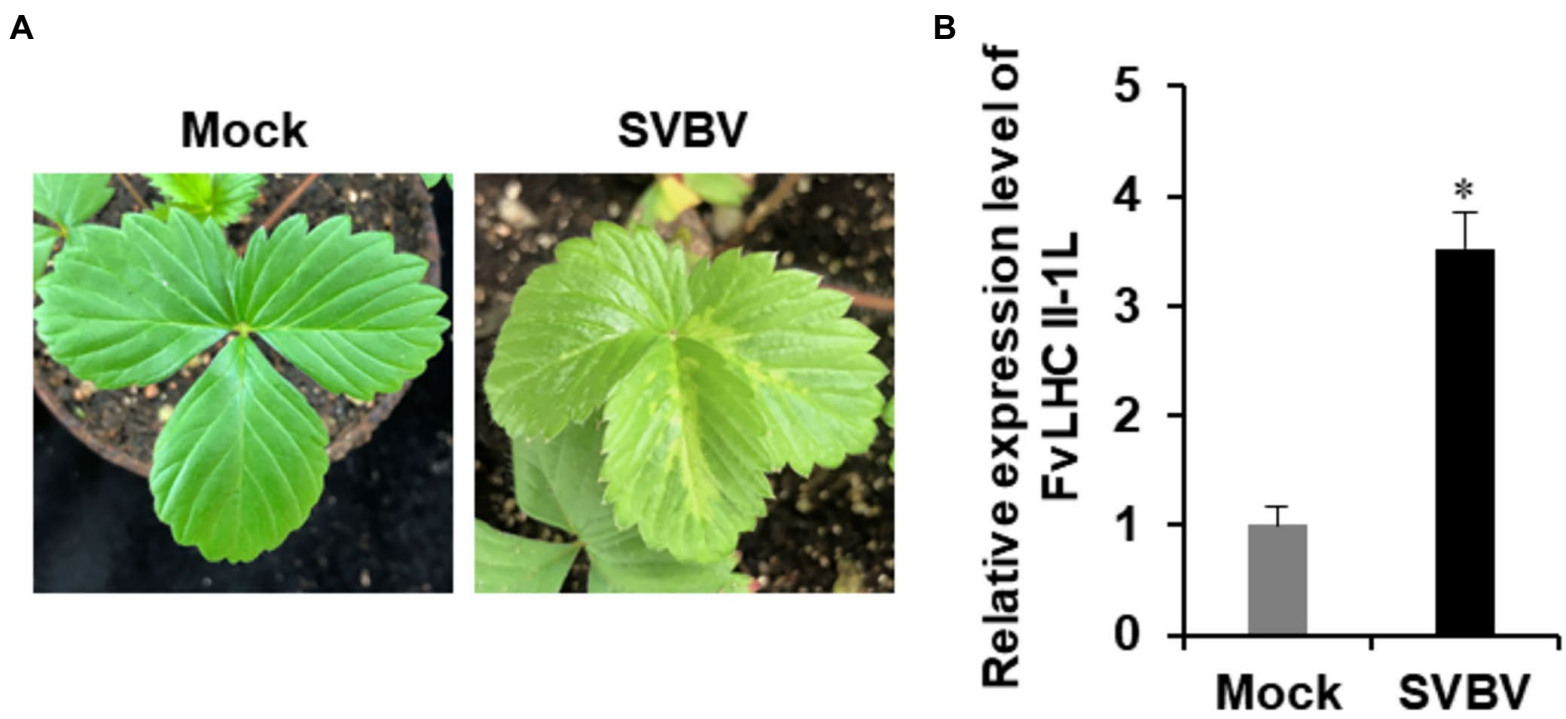
Expression of *FvLHC II-1L* is upregulated by SVBV in infected *F. vesca*. **(A)** Phenotype of mock-inoculated or SVBV-inoculated *F. vesca*. **(B)** The transcript levels of *FvLHC II-1L* in mock-inoculated and SVBV-infected leaves of *F. vesca* were determined using quantitative real-time polymerase chain reaction (RT-qPCR) analysis. Error bars indicate the mean ± standard deviation of three replicates. A two-sample unequal variance directional *t*-test was used to test the significance of the difference (^*^p < 0.05).

To investigate the response of FvLHC II-1L to SVBV infection, healthy *F. vesca* was inoculated with *A. tumefaciens* containing the infectious clone of SVBV using the vacuum-infiltration method, as described previously (Tian et al., 2015). Compared with the mock control, *F. vesca* inoculated with the infectious clone of SVBV showed vein banding symptoms at 35 dpi (Figure 3A). Next, leaves with vein banding symptoms were collected from infected plants and subjected to RT-qPCR analysis. As shown in Figure 3B, the expression of *FvLHC II-1L* was significantly upregulated by approximately 3.5-fold following SVBV infection. This result suggests that SVBV can upregulate the transcript levels of *FvLHC II-1L* in *F. vesca*.

### FvLHC II-1L Assists SVBV P1 to Complement Movement-Defective PVX

**Figure 4:**
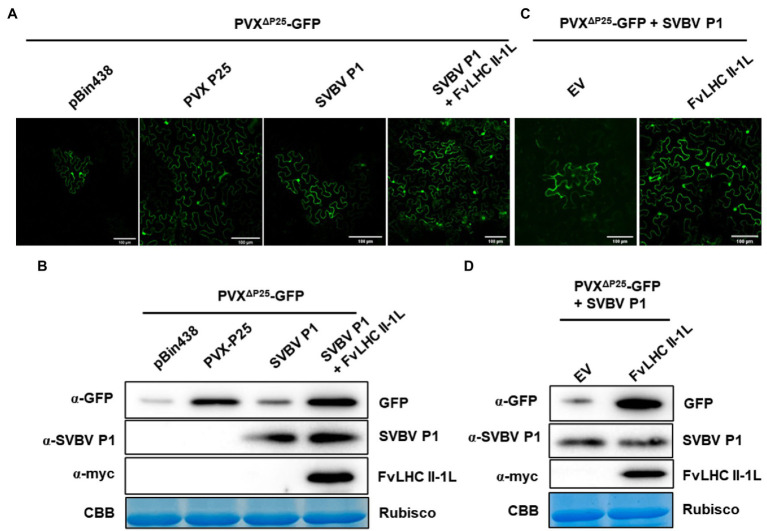
FvLHC II-1L assists SVBV P1 in complementing movement-defective potato virus X (PVX). **(A)** FvLHC II-1L promotes SVBV P1 to complement movement-defective PVX (PVX^ΔP25^-GFP) in *N. benthamiana*. PVX^ΔP25^-GFP was co-infiltrated with pBin438 empty vector, P25, SVBV P1, or SVBV P1 and FvLHC II-1L in *N. benthamiana*. GFP fluorescence was observed at 3 dpi. Bar scale = 100 μm. **(B)** Western blot analysis of GFP, SVBV P1, or FvLHC II-1L protein accumulation in infiltrated leaves at 3 dpi. α-GFP, α-SVBV P1, and α-myc antibodies were used to detect the accumulation of GFP, SVBV P1, and FvLHC II-1L, respectively. Coomassie Brilliant Blue-stained RuBisCO was used as the loading control. **(C)** FvLHC II-1L promotes SVBV P1 to complement PVX^ΔP25^-GFP in FvLHC II-1L transgenic *N. benthamiana*. PVX^ΔP25^-GFP was co-expressed with SVBV P1 in empty vector (EV) or FvLHC II-1L transgenic *N. benthamiana*. GFP fluorescence was observed at 3 dpi. **(D)** The accumulation of GFP, SVBV P1, and FvLHC II-1L proteins in infiltrated leaves was detected *via* western blotting at 3 dpi.

Protein P1 of strawberry vein banding virus has been shown to complement the cell-to-cell movement of movement-defective PVX (Rui et al., 2021). To determine whether FvLHC II-1L affects the function of SVBV P1, we performed a complement assay. *A. tumefaciens* containing pBin438, P25, pBin438-SVBV P1, and pBin438-MYC-FvLHC II-1L were co-infiltrated with movement-defective PVX (PVX^ΔP25^-GFP) into *N. benthamiana* leaves. At 3 dpi, strong GFP fluorescence was observed in infiltrated and adjacent cells of *N. benthamiana* leaves co-expressing pBin438-FvLHC II-1L and pBin438-SVBV P1, which was stronger than that observed in *N. benthamiana* leaves infiltrated with SVBV P1 alone (Figure 4A). *N. benthamiana* leaves infiltrated with pBin438 or P25 were used as negative and positive controls, respectively (Figure 4A). The accumulation of GFP protein in *N. benthamiana* leaves (Figure 4A) was also determined by western blot analysis. As expected, higher GFP protein accumulation was observed in *N. benthamiana* leaves co-expressing FvLHC II-1L and SVBV P1, compared to *N. benthamiana* leaves infiltrated with pBin438, P25, or pBin438-SVBV P1 (Figure 4B).

To further understand the role of FvLHC II-1L, with respect to SVBV P1 complementing PVX^ΔP25^-GFP, transgenic *N. benthamiana* plants overexpressing MYC-tagged FvLHC II-1L (p1307-MYC-FvLHC II-1L) were generated. The mRNA and protein levels of FvLHC II-1L in transgenic plants were quantified *via* RT-qPCR and western blotting, respectively (Supplementary Figure S1). Then, *A. tumefaciens* containing pBin438-SVBV P1 and PVX^ΔP25^-GFP was co-infiltrated into FvLHC II-1L transgenic *N. benthamiana* leaves. As shown in Figure 4C, the area of GFP fluorescence in FvLHC II-1L transgenic plant leaves was noticeably larger than that in empty vector transgenic *N. benthamiana* plant leaves. Western blot analysis revealed a greater accumulation of GFP protein in FvLHC II-1L transgenic plant leaves, compared to leaves of the empty vector control plant (Figure 4D). Collectively, these results suggest that FvLHC II-1L can assist SVBV P1 in complementing movement-defective PVX.

### FvLHC II-1L Assists SVBV P1 to Complement Movement-Defective CMV

**Figure 5:**
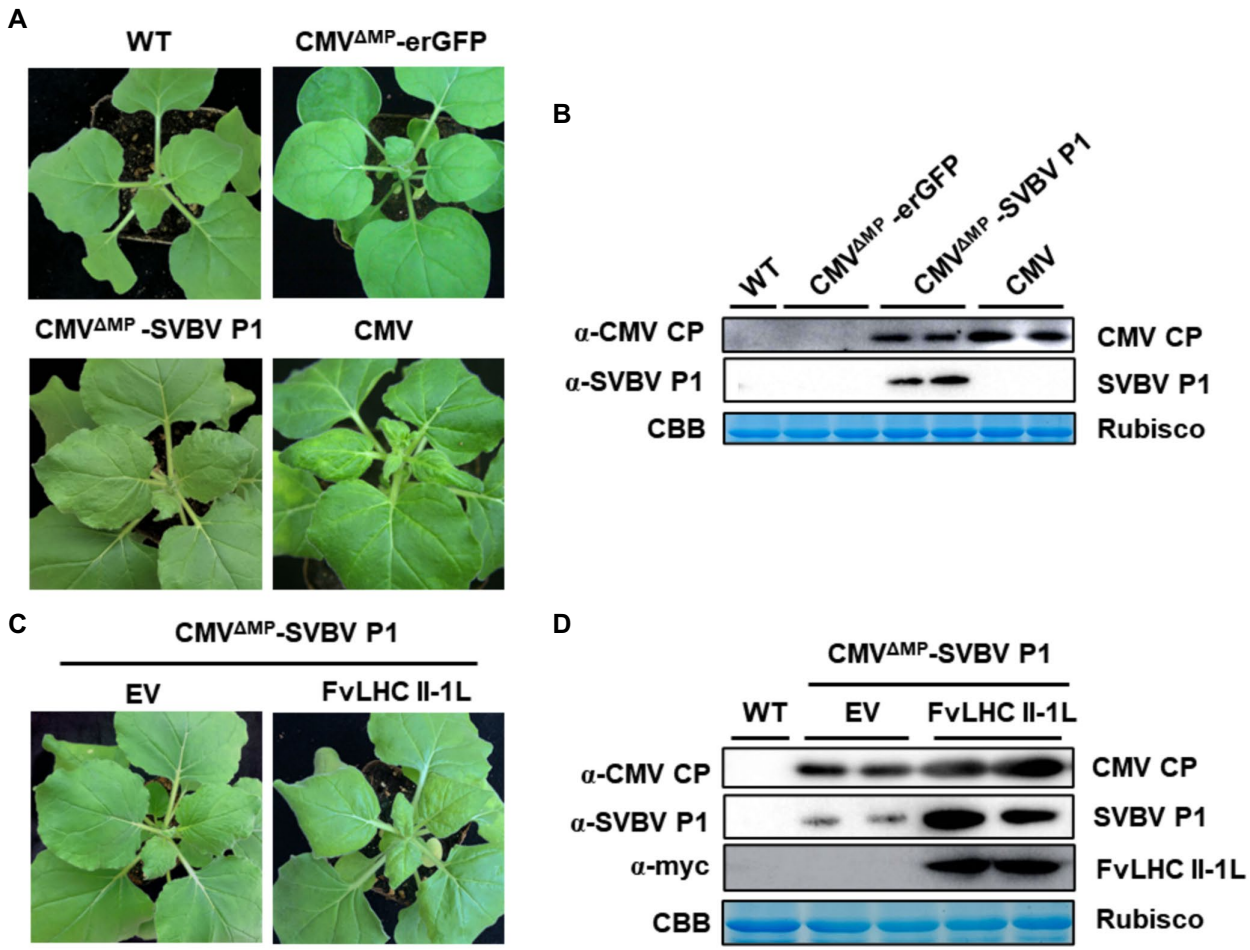
FvLHC II-1L assists SVBV P1 in complementing movement-defective cucumber mosaic virus (CMV). **(A)** Systemic symptoms of wild-type *N. benthamiana* plants agroinfiltrated with CMV, CMV^ΔMP^-erGFP, or CMV^ΔMP^-SVBV P1 at 8 dpi. WT indicates non-infiltrated plants that served as the negative control. **(B)** Accumulation of CMV coat protein (CP) and SVBV P1 in systemically infected leaves was detected *via* western blotting. **(C)** Systemic symptoms of empty vector (EV) or FvLHC II-1L transgenic *N. benthamiana* plants agroinfiltrated with CMV^ΔMP^–SVBV P1 at 8 dpi. **(D)** Accumulation of CMV CP, SVBV P1, and FvLHC II-1L in systemically infected leaves was detected *via* western blotting.

To investigate whether SVBV P1 can complement the systemic movement of CMV^ΔMP^, the recombinant plasmid CMV^ΔMP^-SVBV P1 was constructed. The construct was then transformed into *A. tumefaciens*, followed by infiltration into *N. benthamiana* seedlings. After 8 days, compared with the CMV plant (positive control), more mild leaf crimping was observed in systemic leaves of *N. benthamiana* seedlings infiltrated with CMV^ΔMP^-SVBV P1, while the systemic leaves of CMV^ΔMP^-erGFP-infected *N. benthamiana* seedlings showed no symptoms (Figure 5A). As shown in Figure 5B, the accumulation of CMV CP was detected in CMV^ΔMP^-SVBV P1-infected *N. benthamiana* systemic leaves but not in CMV^ΔMP^-erGFP-infected *N. benthamiana* systemic leaves.

To determine whether FvLHC II-1L promotes SVBV P1 to complement the movement of CMV^ΔMP^, empty vector and FvLHC II-1L transgenic *N. benthamiana* leaves were infiltrated with CMV^ΔMP^-SVBV P1. Compared with the empty vector plants, more severe symptoms were observed in the systemic leaves of CMV^ΔMP^-SVBV P1-infected FvLHC II-1L transgenic *N. benthamiana* (Figure 5C). As expected, CMV CP and SVBV P1 protein accumulation was higher in FvLHC II-1L plants (Figure 5D). These results suggest that SVBV P1 was able to complement the systemic movement of CMV^ΔMP^, and FvLHC II-1L can enhance the complement function of SVBV P1.

### Overexpression of FvLHC II-1L Promotes *Fragaria vesca* Infection by SVBV

**Figure 6:**
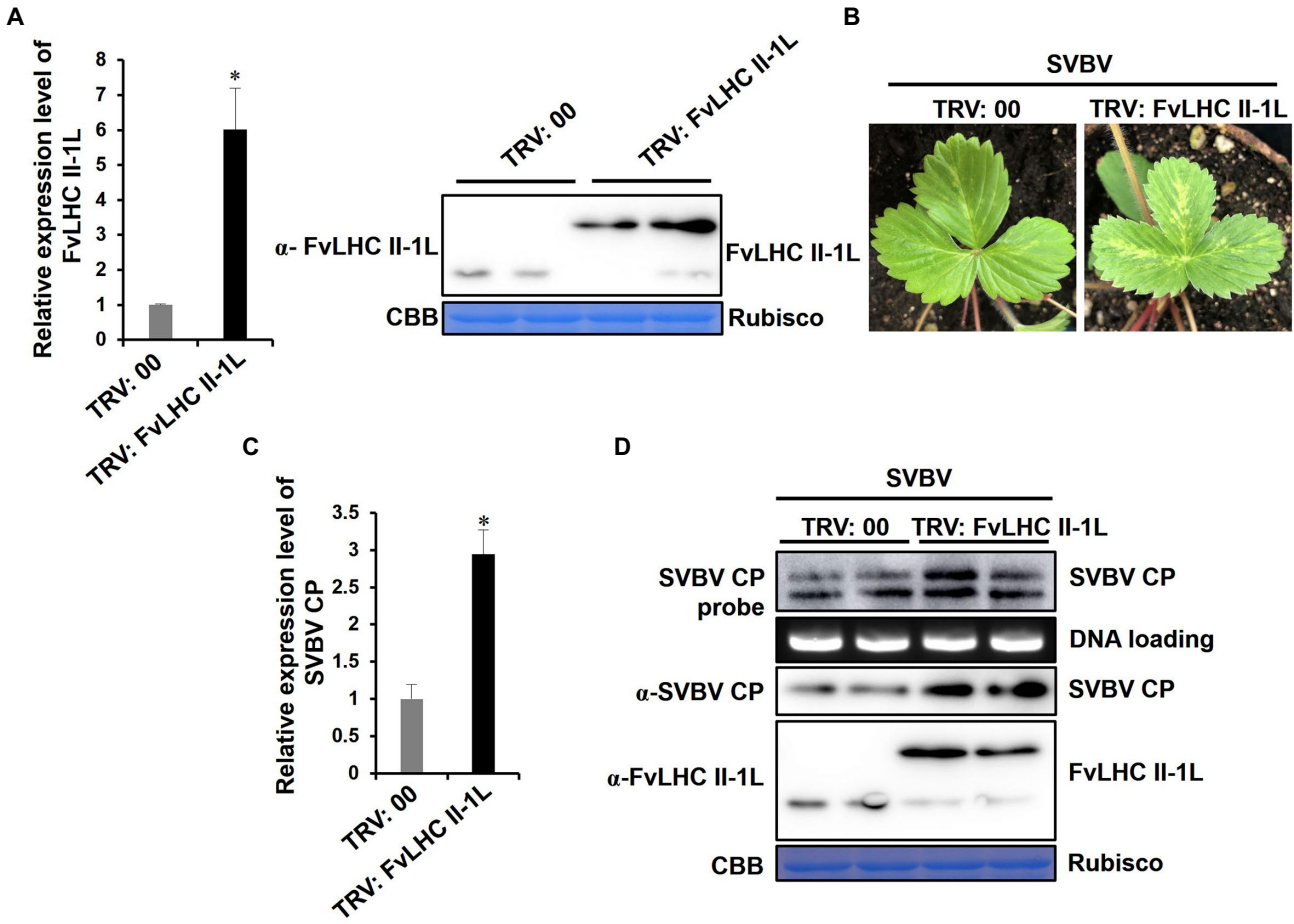
Overexpression of FvLHC II-1L promotes *F*. *vesca* infection by SVBV. **(A)** The transcript levels of *FvLHC II-1L* and the accumulation of FvLHC II-1L protein in TRV:00-infected or TRV:FvLHC II-1L-infected leaves of *F. vesca* were determined *via* RT-qPCR analysis and Western blot at 15 dpi. TRV:00 indicates plants infiltrated with tobacco rattle virus (TRV) empty vector, serving as the negative control. **(B)** Systemic symptoms of TRV:00 or TRV:FvLHC II-1L pre-infected plants inoculated with SVBV *via* vacuum-infiltration. The phenotype of SVBV infection was monitored and photographed at 35 days post SVBV inoculation. **(C)** The transcript levels of *SVBV CP* in systemically infected leaves of TRV:00 and TRV:FvLHC II-1L pre-infected *F*. *vesca* were quantified using RT-qPCR analysis. **(D)** Accumulation of SVBV CP and FvLHC II-1L proteins and SVBV CP DNA, in systemically infected leaves, was detected *via* Western and Southern blotting, respectively.

To further clarify the role of FvLHC II-1L in the infection progression of SVBV in *F. vesca* plants, we used the TRV system to overexpress’in *F*. *vesca* seedlings before inoculation with SVBV. At 15 dpi, systemic leaves of *F. vesca* seedlings were sampled and subjected to RT-qPCR and western blot analyses. The results showed that the transcript levels of *FvLHC II-1L* and the accumulation of CP-FvLHC II-1L fusion protein in *F. vesca* seedlings infiltrated with pTRV-FvLHC II-1L were higher than those in the seedlings infiltrated with TRV:00 (negative control; Figure 6A).

At 15 dpi, TRV:00-infiltrated or TRV:FvLHC II-1L-infiltrated plants were infiltrated with the infectious clone of SVBV. After 35 days, the systemic symptoms of TRV:FvLHC II-1L-infiltrated plants were more severe than those of TRV:00-infiltrated plants (Figure 6B). As expected, RT-qPCR, western blotting, and Southern blotting showed that the levels of SVBV CP mRNA transcripts, SVBV CP protein, and DNA accumulation were significantly increased in TRV:FvLHC II-IL plants (Figures 6C,D). These results indicated that FvLHC II-1L promoted *F. vesca* infection by SVBV.

## Discussion

To infect host plants, plant viruses have to move between cells to achieve local infection, and then move further, over longer distances through the phloem vascular bundles to achieve systematic infection. Both these processes are related to MPs encoded by viruses (Zhou et al., 2019). Not only is SVBV P1 essential for SVBV intercellular movement but it can also complement the movement function of heterologous viruses and promote the intercellular movement of movement-deficient PVX (Rui et al., 2021). In this study, we found that compared with the movement-deficient CMV, the movement-deficient CMV inserted with SVBV P1 systematically moved to the upper leaves *via* long-distance movement, indicating that SVBV P1 complemented the movement-deficient CMV (Figures 5A,B). This complement function is not unique to SVBV P1. The MP Nsvc4 encoded by RSV can complement cell-to-cell movement of movement-deficient PVX (Fu et al., 2018). The MP NSm, encoded by tomato spotted wilt virus, can complement cell-to-cell and long-distance movement of movement-deficient tobacco mosaic virus (TMV) and CMV (Li et al., 2009). The MPs encoded by some Rhabdoviruses can complement the intercellular movement of movement-deficient tomato mosaic virus and PVX (Zhou et al., 2019). Thus, the functions of MPs of different viruses are complementary.

During viral infection, many proteins encoded by plant viruses can interact with chloroplast proteins and affect the course of disease development (Bhat et al., 2013; Mathioudakis et al., 2013; Cheng et al., 2021). The replicase protein of TMV interacts with the chloroplast ATPase *γ* subunit AtpC to block ATP synthesis, decrease photosynthate accumulation, and aggravate viral symptoms (Bhat et al., 2013). The replicase protein of tobacco vein banding mosaic virus interacts with the chloroplast 50S ribosomal protein large submit 1 to promote the accumulation of viral replicase and improve viral replication (Cheng et al., 2021). Pepino mosaic virus (PepMV) MP P26 interacts with tomato chloroplast catalase 1 (CAT1) to improve the activity of CAT1. Silencing of the *CAT1* gene of *N. benthamiana* resulted in a significant decrease in RNA accumulation of PepMV and inhibition of systemic infection by the virus (Mathioudakis et al., 2013). These results suggest that host chloroplast proteins have a close association with plant viral infection. In the present study, the FvLHC II-1L protein, which is another chloroplast protein responsible for maintaining the stability of the electron transport chain of PS II, is an important regulatory factor of photosynthesis and participates in normal plant growth and development. In a previous study, we screened the FvLHC II-1L protein from the cDNA library of *F. vesca* using SVBV P1 as the bait protein (Zhang et al., 2019). Here, FvLHC II-1L was shown to interact with SVBV P1 both *in vitro* and *in vivo* (Figure 1). Further experiments showed that the transcription of *FvLHC II-1L* was upregulated during SVBV infection, suggesting that FvLHC II-1L may be involved in viral infection (Figure 3). This finding is consistent with those of previous studies showing that chloroplast proteins frequently have important roles in facilitating viral infection (Cheng et al., 2021).

Furthermore, we noted that SVBV P1 and FvLHC II-1L were co-located at the edge of cells, wherein SVBV P1 formed irregular aggregates (Figure 2). FvLHC II-1L not only assists SVBV P1 to accelerate the intercellular movement of the PVX transport-deficient mutant but also promotes SVBV P1 to complement the systemic movement of the CMV transport-deficient mutant (Figures 4, 5). It has been reported that the MP P3N-PIPO of turnip mosaic virus (TuMV) interacts with *Arabidopsis thaliana* plasma membrane-associated Ca^2+^ binding protein 1 to expand the diameter of PD and promote viral infection (Vijayapalani et al., 2012). The PVX MP TGB12K interacts with tobacco TIP1 protein, which can act on β-1,3-glucanase and increase the diameter of PD, thereby promoting intercellular transport of PVX (Fridborg et al., 2003). The P25 protein of PVX interacts with the chloroplast ferridoxin 1 (FD1) protein to reduce callose deposition in PD and enhance viral infection (Yang et al., 2020). Rice chloroplast FD1 has previously been demonstrated to be an important component of the photosynthetic electron transport chain that can affect plant growth and development. Therefore, it is reasonable to speculate that FvLHC II-1L functions as a key protein in the photosynthetic electron transport chain of *F. vesca*, which can interact with SVBV P1 and co-locate with it in cell walls, decrease callose deposition, increase the diameter of PD, and promote viral intercellular and systemic movement. Additionally, in the current study, we showed that overexpression of FvLHC II-1L accelerated the course of SVBV infection (Figure 6).

Chloroplasts are not only involved in photosynthesis but also have a close association with salicylic acid and other resistance pathways that are activated by the accumulation of reactive oxygen species (ROS; Nomura et al., 2012; Chan et al., 2016; Serrano et al., 2016). The C4 protein encoded by tomato yellow leaf curve virus can move from the cell membrane to the chloroplast during tobacco infection and can bind to the calcium sensor protein of chloroplasts to inhibit resistance signal transduction (Medina-Puche et al., 2020). The γb protein, encoded by BSMV, interacts with chloroplast NADPH-dependent thioredoxin reductase C (NTRC) to inhibit NTRC-mediated antioxidant defense and promote viral infection (Wang et al., 2021). Similarly, the VPg protein of TuMV interacts with the NADH dehydrogenase-like complex M subunit (NdhM) of *N. benthamiana* chloroplasts to prevent export from the nucleus and inhibit the NdhM-mediated defense response in the host (Zhai et al., 2021). FvLHC II-1L is the core protein of the light-harvesting complex of PS II in *F. vesca*. We speculated that the overexpression of FvLHC II-1L would result in chloroplasts absorbing excess light energy, leading to disruption of the balance between ROS production and cleanup and initiation of ROS-dependent resistance signal pathways. Interaction of SVBV P1 with FvLHC II-1L likely reduces the levels of FvLHC II-1L in chloroplasts, leading to a reduction in the intensity of photosynthesis, and subsequently, the reduction of ROS accumulation, inhibition of immune response, and promotion of viral infection.

## Data Availability Statement

The original contributions presented in the study are included in the article/Supplementary Material; further inquiries can be directed to the corresponding authors.

## Author Contributions

XZ, LJ, and TJ conceived and designed the study. SX, XZ, KX, and ZW performed the experiments and data analysis. SX, XZ, and KX wrote the manuscript. All authors contributed to the article and approved the submitted version.

## Funding

This research was funded by the Grants from the National Natural Science Foundation of China (nos. 32072386 and 31801700), the Anhui Provincial Key Research and Development Plan (no. 202004a06020013), the Zhejiang Basic Public Welfare Research Project of China (no. LGN19C140002), and the Postdoctoral Science Foundation of Anhui Province (no. 2019B360).

## Conflict of Interest

The authors declare that the research was conducted in the absence of any commercial or financial relationships that could be construed as a potential conflict of interest.

## Publisher’s Note

All claims expressed in this article are solely those of the authors and do not necessarily represent those of their affiliated organizations, or those of the publisher, the editors and the reviewers. Any product that may be evaluated in this article, or claim that may be made by its manufacturer, is not guaranteed or endorsed by the publisher.

## Abbreviations

*A. tumefaciens*: *Agrobacterium tumefaciens*
AMV: Alfalfa mosaic virus
BiFC: Bimolecular fluorescence complementation
BSMV: Barley stripe mosaic virus
CMV^ΔMP^: Movement-defective cucumber mosaic virus
CAT1: chloroplast catalase 1
cDNA: Complementary DNA
CP: Coat protein
dpi: Days post inoculation
erGFP: Endoplasmic reticulum-targeted green fluorescent protein
EV: Empty vector
FD1: Ferridoxin 1 protein
*F. vesca*: *Fragaria vesca*
FvLHC II-1L: Chlorophyll a/b-binding protein of light-harvesting complex II type 1 like of *Fragaria vesca*
GFP: Green fluorescent protein
LHC II: Light-harvesting complex II
MP: Movement protein
*N. benthamiana*: *Nicotiana benthamiana*
NdhM: NADH dehydrogenase-like complex M subunit
NTRC: NADPH-dependent thioredoxin reductase C
PD: Plasmodesmata
PepMV: Pepino mosaic virus
PSII: Photosystem II
PVX^ΔP25^-GFP: Movement-deficient potato virus X green fluorescent protein
RT-qPCR: Quantitative real-time polymerase chain reaction
REM1: Remorin protein
RFP: Red fluorescent protein
ROS: Reactive oxygen species
RSV: Rice stripe virus
SVBV: Strawberry vein banding virus
SVBV P1: Movement protein P1 of strawberry vein banding virus
TMV: Tobacco mosaic virus
TRV: Tobacco rattle virus
YFP: Yellow fluorescent protein
Y2H: Yeast two-hybrid
