# Supplementary material for: Strawberry Vein Banding Virus Movement Protein P1 Interacts With Light-Harvesting Complex II Type 1 Like of Fragaria vesca to Promote Viral Infection
Source: Front Microbiol. 2022 May 26;13:884044. doi: 10.3389/fmicb.2022.884044 (PMC9201980; doi:10.3389/fmicb.2022.884044)
Supplement: Supplementary file 1 [file Table_1.DOCX]

**Table S1: Sequence of primers used for PCR amplifications**

| Primer | Nucleotide sequence (5′-3′) | Uses |
| --- | --- | --- |
| FvLHC II-1L-RFP-1-F (*Kpn* I) | CTTTCGCGAGCTCGGTACCATGGCTGCTTCAACAATG | Used to construct pCAM2300-FvLHC II-1L-RFP |
| FvLHC II-1L-RFP-1-R | GTTCTCGGAGGAGGCCATCTTTCCGGGAACAAAGTTG |  |
| FvLHC II-1L-RFP-2-F | CAACTTTGTTCCCGGAAAGATGGCCTCCTCCGAGAAC |  |
| FvLHC II-1L-RFP-2-R (*Xba* I) | CCTGCAGGTCGACTCTAGACTACAGGAACAGGTGG |  |
| nYFP-FvLHC II-1L-F (*Sal* I) | ATCGAGGACTCCGGAGTCGACATGGCTGCTTCAACAATGGC | Used to construct pCAM1300-nYFP-FvLHC II-1L |
| nYFP-FvLHC II-1L-R (*Sma* I) | TGGGGATCCGCATCTCCCGGGTCACTTTCCGGGAACAAAG |  |
| FvLHC II-1L-Flag-F (*Bam*H I) | GGATCCATGGCTGCTTCAACAATGGCTCTG | Used to construct pCAM1307-FvLHC II-1L-Flag |
| FvLHC II-1L-Flag-R (*Sal* I) | GTCGACTCACTTTCCGGGAACAAAGTTGGTG |  |
| FvLHC II-1L-Q-F | ACCGTGTCAAGTACTTGG | Used to construct RT-qPCR analyze |
| FvLHC II-1L-Q-R | CTTCTCCGAACTTGACTC |  |
| TRV2-LHC II-1L-1-F(*Hin*d III) | TTGGGCCCGGCGCGCCAAGCTTGCATGCCTGCAGGTC | Used to construct pTRV: FvLHC II-1L |
| TRV2-LHC II-1L-1-R | GCCATTGTTGAAGCAGCCATGGGATTAGGACGTATCGG |  |
| TRV2-LHC II-1L-2-F | TCCGATACGTCCTAATCCCATGGCTGCTTCAACAATGGC |  |
| TRV2-LHC II-1L-2-R | GAGTTCACGTCCTTAAATCCTCACTTTCCGGGAACAAAGT |  |
| TRV2-LHC II-1L-3-F | ACTTTGTTCCCGGAAAGTGAGGATTTAAGGACGTGAACTC |  |
| TRV2-LHC II-1L-3-R(*BamH* I) | CGTGAGCTCGGTACCGGATCCCCATGGAGGCCTTCT |  |
| MYC-LHC II-1L-F (*Xba* I) | GACTTGAACTCGGTATCTAGAATGGCTGCTTCAACAATG | Used to construct pCAM1307-MYC-FvLHC II-1L |
| MYC-LHC II-1L-R (*Hin*d III) | GTCGACGGTATCGATAAGCTTTCACTTTCCGGGAACAAAG |  |
| cYFP-P1-F (*Sal* I) | CTGTACAAGTCCGGAGTCGACATGTCTGAAGAAGAAATCAG | Used to construct pCAM1300-cYFP-  SVBV P1 |
| cYFP-P1-R (*Sma* I) | TGGGGATCCGCATCTCCCGGGCTATAGCTGATGTGCAAGGGTAT |  |
| P1-GFP-1-F (*Kpn* I) | TACGAATTCGAGCTCGGTACCATGTCTGAAGAAGAAATC | Used to construct pCAM2300-SVBV P1-GFP |
| P1-GFP-1-R | GTTCTTCTCCTTTACTCATTAGCTGATGTGCAAG |  |
| P1-GFP-2-F | CTTGCACATCAGCTAATGAGTAAAGGAGAAGAAC |  |
| P1-GFP-2-R (*Xba* I) | ATGCCTGCAGGTCGACTCTAGATTAAAGCTCATCATGTTTG |  |
| CMV RNA3^ΔMP^-P1-1-F  (*Nco* I) | CCATGGATGTCTGAAGAAGAAATCAG | Used to construct pCB301-CMV RNA3^ΔMP^-P1 |
| CMV RNA3^ΔMP^-P1-1-R  (*Xba* I) | TCTAGACTATAGCTGATGTGCAAGGG |  |
| SVBV CP-Q-F | CTATCGTCCGAACCGAAAG | Used to construct RT-qPCR analyze |
| SVBV CP-Q-R | GGCATTACCAACAGTCTTAC |  |
| SVBV CP-S-F | AGAGAAAGACTCGAACAACTG | Used to construct Southern blot |
| SVBV CP-S-R | CTTCAATGAGTTGTCTTCGG |  |
| CFP-F(*BamH* I) | GGATCCATGGTGAGCAAGG | Used to construct pCAM1307-CFP-Flag |
| CFP-R(*Sal* I) | GTCGACTCACTTGTACAGCTCGTC |  |
| pSVBV P1-F(*BamH* I) | GGATCCATGTCTGAAGAAGAAATCAG | Used to construct pBin438-SVBV P1 |
| pSVBV P1-R  (*Sal* I) | GTCGACCTATAGCTGATGTGCAAGGG |  |
| hSVBV P1-F  (*EcoR* I) | GAATTCATGTCTGAAGAAGAAATCAG | Used to construct pHMTc-SVBV P1 |
| hSVBV P1-R  (*BamH* I) | GGATCCCTATAGCTGATGTGCAAGGG |  |
| pFvLHC II-1L-F(*BamH* I) | GGATCCGAGCAAAAGCTCATTTCTG | Used to construct pBin438-Myc-FvLHC II-1L |
| pFvLHC II-1L-R(*Sal* I) | GTCGACTCACTTTCCGGGAACAAAG |  |
| LHC II-GFP-1-F (*Kpn* I) | TACGAATTCGAGCTCGGTACCATGGCTGCTTCAACAATG | Used to construct pCAM2300-FvLHC II-GFP |
| LHC II-GFP-1-R | GTTCTTCTCCTTTACTCATCTTTCCGGGAACAAAGTTG |  |
| LHC II-GFP-2-F | CAACTTTGTTCCCGGAAAGATGAGTAAAGGAGAAGAAC |  |
| LHC II-GFP-2-R (*Xba* I) | GCCTGCAGGTCGACTCTAGATTAAAGCTCATCATGTTTG |  |
| Nbactin-F | TGGCTCTTGACTACGAGCAGGAGCTT | Used to construct RT-qPCR analyze |
| Nbactin-R | ACCACTGAGCACAATGTTACCGTAGAGGT |  |
| Fvactin-F | TCCCTGGCATTGCCGACAGAATGAG |  |
| Fvactin-R | CCTTTGCAATCCACATCTGCTGGAATG |  |

*-Underlined sequences indicate various restriction sites.
