# Supplementary figures and images for: Strawberry Vein Banding Virus Movement Protein P1 Interacts With Light-Harvesting Complex II Type 1 Like of Fragaria vesca to Promote Viral Infection
Source: Front Microbiol. 2022 May 26;13:884044. doi: 10.3389/fmicb.2022.884044 (PMC9201980; doi:10.3389/fmicb.2022.884044)

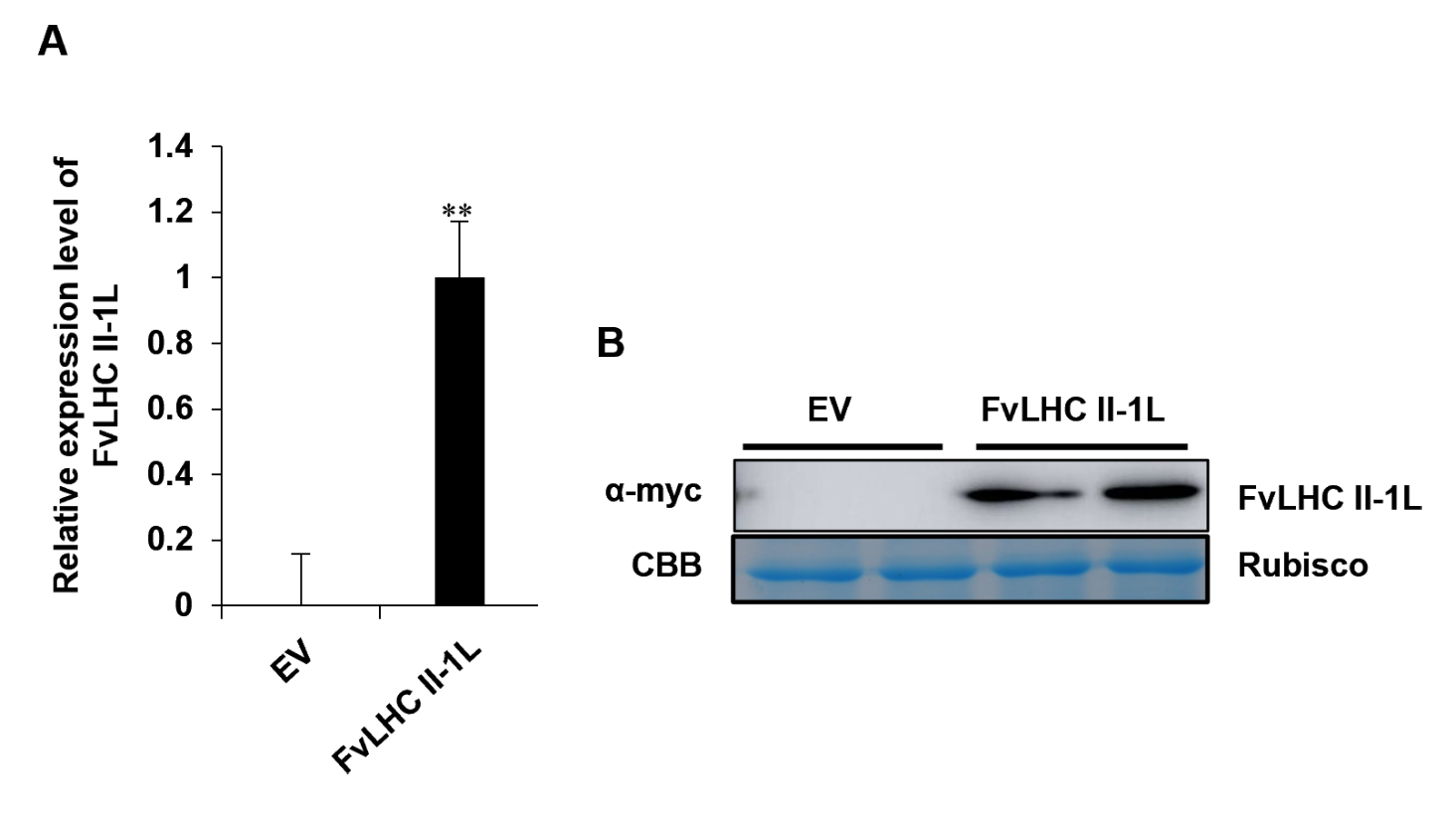

Supplement: Supplementary file 2 [file Image_1.JPEG]
